# The Asenze Cohort Study in KwaZulu-Natal, South Africa: protocol and cohort profile

**DOI:** 10.4178/epih.e2022037

**Published:** 2022-04-05

**Authors:** Chris Desmond, Gabriella A. Norwitz, Jane D. Kvalsvig, Rachel S. Gruver, Shuaib Kauchali, Kathryn G. Watt, Nonhlanhla P. Myeza, Adele Munsami, Leslie L. Davidson

**Affiliations:** 1 Center for Rural Health, University of KwaZulu-Natal, Durban, South Africa; 2 Department of Epidemiology, Mailman School of Public Health, Columbia University, New York, NY, USA; 3 Department of Public Health, University of KwaZulu-Natal, Durban, South Africa; 4 Maternal, Adolescent, and Child Health Institute NPC (MatCH), Durban, South Africa; 5 Department of Pediatrics, Columbia College of Physicians and Surgeons, Columbia University, New York, NY, USA

**Keywords:** Population-based cohort, Low-and-middle income country, South Africa, HIV/AIDS, Child and adolescent health, Child and adolescent development

## Abstract

The Asenze cohort is set in South Africa, a middle-income country impacted by one of the highest global rates of people living with HIV/AIDS and high levels of socioeconomic inequality. This longitudinal population-based cohort of children and their primary caregivers assesses household and caregiver functioning, child health, social well-being, and neuro-development from childhood through adolescence. Almost 1,600 children born at the peak of the human immunodeficiency virus epidemic (2003-2005) were followed (with their primary caregivers) in 3 waves, between 2008 and 2021, at average ages of 5, 7, and 16. Wave 3 is currently underway, having assessed over 1,100 of the original wave 1 children. Wave 4 begins in 2022. The study, with a dyadic structure, uses a broad range of measures, validated in South Africa or recommended for global use, that address physical, social and neuro-development in childhood and adolescence, and the social, health, and psychological status of children’s primary caregivers. The Asenze study deepens our understanding of childhood physical, cognitive, and social abilities and/or disabilities, including risk-taking behaviors, and biological, environmental, and social determinants of health. We anticipate the findings will contribute to the development of community-informed interventions to promote well-being in this South African population and elsewhere.

## INTRODUCTION

This paper outlines the protocol, history, and scope of the Asenze Cohort Study in KwaZulu-Natal, South Africa. South Africa is a middle-income country shaped by the legacies of apartheid, periods of violent political instability, and an initial refusal of the government to acknowledge human immunodeficiency virus (HIV) as the cause of acquired immune deficiency syndrome (AIDS). These historic and ongoing challenges have led to South Africa having one of the highest prevalence rates of people living with HIV/AIDS [1] and one of the highest rates of socioeconomic inequality [2].

The HIV/AIDS epidemic has increased the burden of childhood developmental disability and challenges to adolescent health and well-being in low-income and middle-income countries. HIV/AIDS is a multi-system, chronic illness whose impact on child health is physical, cognitive, and/or psychological. Children whose parent(s) are living with HIV/AIDS may be affected directly, through vertical transmission from mother to child, or indirectly, due to comorbidities or early mortality of a caregiver, which may in turn impact the quality of child care. HIV is a leading cause of disability across South Africa, including KwaZulu-Natal, the province with the highest prevalence of HIV [3]. Given HIV’s impact across the life course, research from birth cohorts such as the Asenze cohort can play an important role in informing international, national, and local strategies to improve child and adolescent outcomes.

### Overview of the timeline

A pilot study between 2003 and 2005 established the feasibility and acceptability of our population-based cohort study using a range of socio-logical and neuro-developmental measures. Implementation of the main population-based cohort study began in 2008, among eligible enrolled children, at an average age of 5, who were assessed again at an average age of 7. Following a gap in contact due to funding constraints, the children with their primary caregivers have been followed up in a third wave to adolescence. The extensive information gathered about children and their primary caregivers over the 3 time points allows both a cross-sectional and a longitudinal analysis of the household situation, the primary caregivers’ functioning, and children’s health, behavior, and neuro-development, with the long-term goal of promoting better physical, cognitive, and psychosocial functioning.

### Study aims and theoretical models

**Figure 1.**
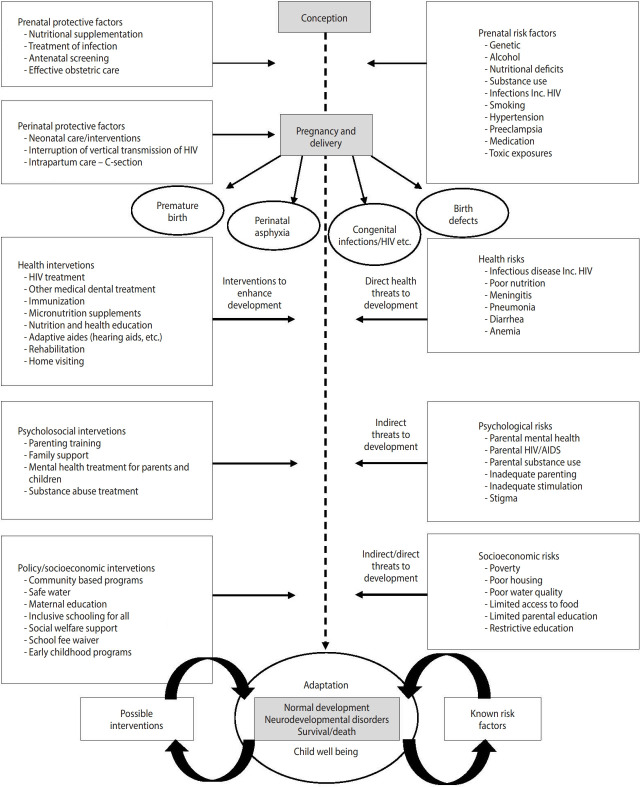
Theoretical model for waves 1 and 2. HIv, human immunodeficiency virus; AIDS, acquired immune deficiency syndrome.

The first 2 waves of the study aimed to assess over time the ability of children to function physically, cognitively and socially. The study focused on how children are influenced by health-related (HIV, anemia, infections), contextual (socioeconomic, environmental, access to care, intervention), and psychosocial (primary caregiver substance use and mental health problems, family functioning) factors. We adopted a theoretical model for waves 1 and 2 [4] similar to models proposed by United Nations Children’s Fund (UNICEF) [5], with the central outcome of child neuro-development (Figure 1).

**Figure 2.**
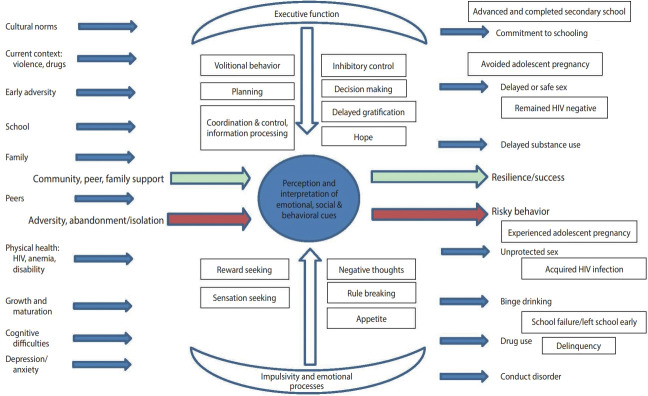
Theoretical model for wave 3. The interaction between ‘top down’ excutive function and ‘bottom up’ impulsive and emotional processes in regulating social, cultural and biological challenges of adolescent development with positive and negative outcomes. HIV, human immunodeficiency virus.

Wave 3 of the study, now nearly complete, aims to examine how early childhood adversities impact ongoing cognitive development, and, through a range of social and biological pathways, result in educational challenges and risky behavior in adolescents. We adopted a socio-ecological model of risk and protection wedded to a life-course perspective (Figure 2) [6,7]. This model investigates, over time, predictors and modifiers of unprotected sex, substance use, binge drinking, and school drop-out. These include individual attributes such as aspects of executive function (impulsivity, planning, attention) and attitudes of hope and future orientation as well as contextual factors (family support, primary caregiver HIV status and mental health, stress, trauma, and exposure to poverty and violence). Wave 4 will assess adolescents 1 year to 2 years later to examine, in particular, school drop-out at this critical time period. Wave 4 will begin in 2022.

## MATERIALS AND METHODS

### Design

The Asenze study is a longitudinal, population-based study. Children between the ages of 4 years and 6 years were identified in a defined population and have been followed up to mid-adolescence. The name Asenze, meaning “let us act” in Zulu, creates a sense of joint agency and collaboration with the participating communities.

### Study area and early steps

KwaZulu-Natal is the second most populous of the 9 provinces of South Africa, with 11 million people. This province had one of the highest HIV/AIDS rates in the world, with 6% of 2-year-old to 9-year-old children being HIV-positive in 2002 [1,8]. Around the year children in the study were born, in 2003, the prevalence of HIV infection in females seeking antenatal maternity care in KwaZulu-Natal was 39.5% [9]. The Asenze study is situated in the Valley of a Thousand Hills, which encompasses both peri-urban and rural communities. The study area is 45 km northwest of Durban, the largest city in the province, and is populated largely by Zulu people. Because of the lack of local transportation, the study provided transportation to and from study offices for all 3 waves.

#### Local consultation and partnership

The pilot study and the subsequent 3 waves of Asenze were led jointly by research teams at the University of KwaZulu-Natal and Columbia University’s Mailman School of Public Health. The study offices and clinic were based at the Valley Trust, a leading non-governmental service organization. The Valley Trust collaborated with Asenze researchers in the pilot study funded by the National Institutes of Health Fogarty’s Global Brain Disorders Research program (discussed below). Subsequent plans for the larger cohort study were discussed with key stakeholders, including the leadership of 5 local authorities of the KwaDedangendlale area, who approved the study and gave valuable input such as prioritizing exploring inappropriate alcohol usage. The study was also discussed with a range of local, district, and national health officials to gain support and solicit suggestions for study aims.

#### Pilot study design and population

The pilot study, funded between 2003 and 2005 by the Brain Disorders Program of the Fogarty Center at the National Institutes of Health, selected 77 households that had a child with a disability and were receiving services from the Valley Trust, creating a convenience sample of 124 children aged 2 years to 9 years. Given the focus on neuro-disability, 19 children living with diagnosed neuro-disabilities and 105 children who were not disabled (often siblings) were enrolled. The pilot was designed to test proposed measures and the feasibility and acceptability of a large cohort study of children and their primary caregivers in the same study areas. The pilot results were not published, but included an association between child disability and maternal depression, the finding that HIV testing of children was acceptable to the families, and the observation that children with even mild neuro-developmental disabilities were less likely to be in school than children of a similar age without neuro-developmental disabilities. The study found acceptable correlations between a cognitive measure validated in South Africa, the Grover Counter Scale, and the McCarthy Scales, leading us to include the Grover Counter Scale in waves 1 and 2 alongside the sub-scales of the Kaufman Assessment Battery for Children, as it was more widely used at that time in sub-Saharan Africa.

### Measures, validation, and translation in the main Asenze Cohort Study

The Asenze study prioritized the use of validated measures and, when possible, measures validated in South Africa (Table 1). All wave 1 and wave 2 measures not available in Zulu were carefully translated and back translated by 2 team members bilingual in English and Zulu and reviewed by a senior study investigator. Difficulty in translating the Strengths and Difficulties questionnaire led to a close review, the creation of a focus group, and a consultation with a linguist. For wave 3 measures, we adopted a modified committee approach with experienced bilingual translation committee members [10].

### Study population

Five contiguous areas in the Valley of a Thousand Hills were included in this study. The Valley Trust’s prior geographic information systems mapping of all area households provided the sampling frame. In 2008, the study population was established through a door-to-door field survey visiting every household in the 5 local authority areas. All children between the ages of 4 and 6 were eligible to participate in the cohort along with their primary caregivers. Qualitative interviews confirmed by quantitative data found that there was 1 person mainly responsible for making decisions and providing shelter, food, and daily care for the child, and these became the criteria for identifying the primary caregiver, generally a parent, grandparent, or another adult in the household.

### Study structure including measures, recruitment, and retention by wave

**Figure 3.**
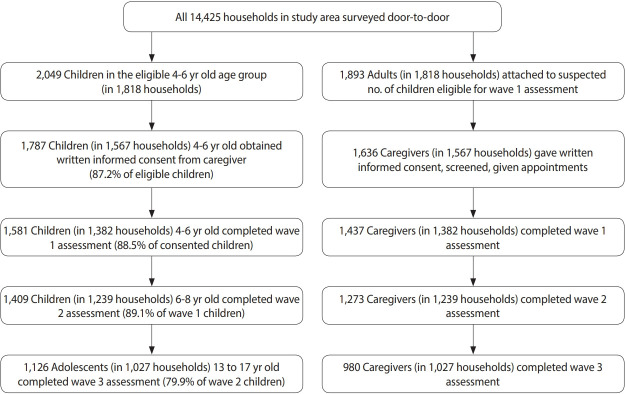
Flow chart of the population over time.

Figure 3 provides the number of participants, both children and primary caregivers, for recruitment and retention.

#### Initial door-to-door field survey

All households in the study area were visited by the team of fieldworkers in 2008. We identified 2,049 eligible children within the study age range living in 1,818 households and looked after by 1,893 primary caregivers. The primary caregivers provided informed consent to obtain household information and completed the Ten Questions, a developmental screening tool for serious child disability validated in this population, on 1,787 children aged 4 years to 6 years (87.2% of eligible children) in 1,567 households. The age range of 4 years to 6 years was selected because the initial focus of the cohort was on emerging neuro-disability, and this age range would allow for a substantive assessment before and after the children entered school. The majority of caregivers provided data on birthweight and vaccination records from the children’s “Road to Health” cards. We were funded for a total of 5 years, which allowed us to document changes before and after schooling began. The caregivers were also asked about household socio-demographic characteristics, employment, and education [11,12]. All consenting primary caregivers received follow-up appointments for the wave 1 assessment approximately 2 weeks later.

#### Wave 1 assessment

Of the 1,787 children with field data on cognitive development and household characteristics, 1,581 children (88.5%) and 1,437 primary caregivers completed the wave 1 assessments between 2008 and 2010. Children who were assessed in wave 1 were statistically significantly more likely to reside in households with a higher asset index than those who were not assessed in wave 1. The asset index was determined by household information from the initial door-to-door survey (wall material, heating fuel, sanitation facility, radio and TV possession, poultry keeping, etc.). Wave 1 participation was also somewhat higher in children who screened positive on the Ten Questions. The study team (a doctor, a bilingual mid-level psychological assessor, and a health assistant) administered comprehensive medical, psychosocial and developmental assessments of the children with their primary caregiver present. Primary caregivers, interviewed separately, provided social, demographic, and health information about themselves and their participating child (Table 1). Both children and primary caregivers were offered rapid HIV testing with counseling and provided an additional informed consent. Hemoglobin levels were obtained for children. Children and primary caregivers found to have untreated health issues, behavioral/mental health problems, or disabilities were referred to local services for further assessment and care.

#### Wave 2 assessment

Of the 1,581 children who completed wave 1 assessments, 1,409 6-year-old to 8-year-old children (89.1%) and 1,273 primary caregivers completed wave 2 assessments between 2010 and 2014. In slightly under 15% of the dyads, the primary caregiver of the child changed between wave 1 and wave 2. The measures in wave 2 were administered approximately 2 years later and were nearly identical to those administered in wave 1. The medical history and examinations were abbreviated, and 2 additional measures of cognitive achievement were added (Table 1).

#### Wave 3 assessment

Between waves 2 and 3, there was a follow-up telephone call to confirm contact details. All children and primary caregivers who had participated in wave 2 were invited to participate in wave 3, occurring between 2019 and 2021 when children were, on average, 15.85 years old. In total, 1,126 adolescents, ranging from 13 years to 17 years old, and 980 primary caregivers completed wave 3. Due to the expansion of the study purpose from focusing primarily on childhood developmental disabilities and cognitive development to assessing broader elements of adolescent functioning, including risky behaviors, the wave 3 adolescent measures differed markedly from earlier measures, while the primary caregiver measures remained largely unchanged. We also added a digital cognitive subtest, NeuroScreen, which led to a sub-study exploring the feasibility of using NeuroScreen in local schools (Table 1). The onset of the coronavirus disease 2019 (COVID-19) pandemic during wave 3 data collection led to the addition of a qualitative study on the impact of COVID-19 and the addition of a COVID-19 module to the wave 3 assessments. The COVID-19 pandemic and government lockdown caused a substantial pause in data collection in 2020 and slowed the pace of wave 3 assessments in 2020 and 2021 to ensure the safety of the study personnel and study participants.

### Ethics statement

The Asenze Cohort Study received ethical approval for all waves of the study and any modifications from the Biomedical Research Ethics Committee of the University of KwaZulu-Natal and from the Institutional Review Board of Columbia University (IRB No. AAAC2559). Initial approval was also received from local authority councils, the local district health committee, and the local district board of education.

## RESULTS

Tables 2-4 report statistics for the household and family context, as well as child and primary caregiver participants, in waves 1 and 2. A full list of publications and findings is available on the Asenze Cohort Study website (https://crh.ukzn.ac.za/asenze/). Below are summarized findings from waves 1 and 2. Wave 3 data collection was just completed in January 2022, and the results are not yet available.

### Household and family context

There were 1,382 households assessed in wave 1. The household measures (collected in the field in wave 1 only, to be repeated in wave 4) included household composition, employment, education, finances, food insecurity, and other home stressors. Only 8.7% of households had a member with an education past high school, 68.4% relied on social grants, and 23.2% reported having run out of food during the past month. While compiling the household asset index in wave 1, we noted significant differences comparing asset indexes between the 5 contiguous study areas. The primary caregivers from households scoring in the lowest tertile of the index also had a higher rate of HIV and were more likely to have a child with a self-reported disability.

### Children

There were 1,581 children (average age, 5.0 years; range, 3.7 to 6.6) assessed in wave 1 and 1,409 (average age, 6.9 years; range, 5.8 to 8.5) assessed in wave 2. The sex distribution of children was 50/50 in both waves.

#### Child health and behavior

In wave 1, 62 children (3.9%) were reported HIV-positive either by their primary caregiver and/or by an onsite HIV test at the time of assessment. We found that 41% of children with mothers known to be HIV positive had never been tested for HIV, which stemmed from both the health system’s failure to offer comprehensive care and the mothers’ internalized shame, stigma, and subsequent fear of testing their child [13]. Additionally, in wave 1, the child prevalence of lifetime epilepsy was 27/1,000 and that of active epilepsy was 15/1,000. Children with epilepsy were more likely to have behavior problems than children without epilepsy. Moreover, compliance for wave 1 referrals made for non-acute conditions, such as disorders of hearing/middle ear, visual acuity, and anemia, was low, and was influenced by the primary caregiver’s age and household education level and stability [14].

#### Child cognitive outcomes

While studying child cognitive and language outcomes by HIV exposure status in wave 1, Gruver et al. [15] noted that for all outcome measures, HIV-exposed but uninfected children and HIV-unexposed children had comparable scores, while HIV infected children had significantly lower cognitive and language scores. Additionally, in wave 1, Ajayi et al. [16] found that children with low cognitive scores were more often stunted, had no preschool education, and came from areas less favorable in terms of local infrastructure, access to employment, and arable land. Of note, it was reported that 61.2% of children had attended a crèche or preschool. In 2 subsequent papers on wave 2 data, Ajayi et al. [17,18] found that children’s nutritional status directly predicts cognitive test scores and that children’s age, area of residence, height-for-age, and paternal level of education affected cognitive scores. A longitudinal assessment noted that adverse childhood events reported in wave 1 were independently related to child behavior problems in wave 2 [19].

### Primary caregivers

There were 1,437 primary caregivers (average age, 36.9 years) in wave 1 and 1,273 (average age, 38.9 years) in wave 2. The majority of primary caregivers were mothers (65.6% in wave 1 and 61.8% in wave 2) and approximately 20% were grandmothers. A large portion of primary caregivers self-reported or tested HIV-positive: 26.2% in wave 1 and 31.5% in wave 2.

#### Primary caregiver mental health

A large proportion of primary caregivers screened positive for a mental health disorder: 31.3% in wave 1 and 18.9% in wave 2. For mental health disorder screening, Mellins et al. [20] validated the Client Diagnostic Questionnaire against an assessment by a masters-level clinical psychologist and found 73% sensitivity and 80% specificity. In wave 1, primary caregivers who screened positive for at least 1 mental health psychiatric disorder were more likely to be older, have no individual income, and have less formal education [21]. The presence of a mental health psychiatric disorder in primary caregivers was also associated with lower household employment levels and prior household death of a child [21].

#### Primary caregiver intimate partner violence

A large proportion of primary caregivers had ever experienced intimate partner violence (IPV): 43.1% in wave 1 and 46.2% in wave 2. In wave 1, the primary caregiver experiencing IPV was associated with the presence of their child having a behavioral difficulty [22]. Furthermore, in wave 1, 9% of primary caregivers were identified as risky drinkers; risky drinking was associated with IPV as well as smoking, HIV, and caring for a child with a disability [23].

### Engagement with the community

#### Community engagement

The Asenze Study leaders met with the 5 local authorities, the local health officials, and provincial health agencies to secure support and solicit input for wave 1 assessments. Following wave 2, community feedback meetings were held with each of the 5 local authorities. All community members, not only the families of Asenze participants, were invited. The day-long workshops reported the Asenze Study health and neuro-developmental findings, discussed the importance of early nutrition and cognitive stimulation, and included a presentation from the Valley Trust on their proposed early childhood development intervention to address these issues. The meetings were well-attended, with lively question-and-answer sessions.

#### The Valley Trust intervention based on waves 1 and 2

The Valley Trust based their Child Health and Development Program, the Khulakahle Mntwana Program (“the program for children to grow well”), on the findings of the first 2 waves of the Asenze Study [24]. The program, still continuing 8 years later, consists of group work (both male’s and female’s groups) and home visits to families in need. It also focuses on crop and animal production, income generation/savings management, and literacy training, and supports child nutrition and illness needs, as well as primary caregiver psychological distress [24].

#### Asenze learning feasibility study

There are few educational psychologists in South Africa, particularly in the public sector, and a large backlog of children referred for cognitive assessment of learning needs. Working with the approval of the District Department of Basic Education and with assistance from the University of KwaZulu-Natal Discipline of Psychology, as well as 2 local secondary schools, the Asenze team is completing a feasibility study on the use of NeuroScreen, an electronic tablet-based screen comprising 10 brief neuropsychological tests, to assist schools in assessing learners in need of academic assistance [25]. NeuroScreen can be administered by trained staff with hopes of reducing the backlog of learners needing expert assistance.

## KEY FINDINGS

The Asenze Cohort Study is one of a limited number of population-based cohort studies in both sub-Saharan Africa and internationally in low-income and middle-income countries. Moreover, it reaches communities in under-resourced and remote parts of the Valley of a Thousand Hills in KwaZulu-Natal, unlike many studies that only included urban or peri-urban areas. We have achieved a high level of retention, comparable to those in high-income countries. It is important to maintain this cohort as adolescents become adults and face the dual impact of HIV and COVID-19, limited access to education and employment, and widespread poverty.

## STRENGTHS AND WEAKNESSES

The Asenze Cohort Study has many strengths. Its first strength is its design. The longitudinal study design of children and their primary caregivers over time allows causal analyses. The population-based nature reduces the possibility of selection bias and allows the study findings to be generalized to the broader KwaZuluNatal population, broadening the reach of possible interventions. Fortunately, only a small group of families initially declined study enrollment and there has been a high rate of retention of children and primary caregivers in waves 1, 2, and 3. Secondly, the Asenze study employed a mixed-methods approach, integrating qualitative analyses into quantitative findings providing cultural context to the quantitative data. For example, mixed methods were used to explore the meaning of peer relationships in young children [20] and to deepen our understanding of the impact of the COVID-19 pandemic on adolescents and their primary caregivers. Thirdly, the study used a comprehensive set of measures of health and psychosocial well-being on both children and their primary caregivers, many of which are validated in South Africa (Table 1). The Asenze study has also contributed to validating 2 study measures in South Africa: Mellins et al. [20] validated the Client Diagnostic Questionnaire instrument used to screen for mental health disorders and performed a principal component analysis of the Strengths and Difficulties Questionnaire, noting that its total difficulties subscale may be a useful screening tool but that its peer relations subscale was not robust in young children [26]. Finally, the study involved the community in initial consultations and continued to share study findings with community members and local, district, and national authorities.

Due to the design of the study, it started when the children were on average 5 years of age and, as a consequence, there are limited data at the time of pregnancy and birth. In addition, due to challenges in obtaining funding, we were not able to launch a wave in middle childhood. This meant that though waves 1 and 2 were in early childhood, there is a gap between the mean ages of 7 and 16. Although study acceptability to the community and retention was high, we have not had the resources to go back to those lost to follow-up over the first 2 waves. Given funding and ethical approval, we will reach out to re-engage that group as the cohort reaches adulthood.

### Future research

In the next year, using data from waves 1, 2, and 3, we will determine the impact of ongoing and de novo social and economic adversity, primary caregiver HIV-positive status, and primary caregiver mental health on the neuro-development and behavior of children in the cohort over time as they reach mid-adolescence. Looking forward, the Asenze team will complete wave 4 to capture the transition from mid- to late adolescence. As the study findings continue to deepen our understanding of children’s physical, cognitive, and social disabilities, adolescent cognition, risk-taking, and education and primary caregivers’ biological, environmental, and social determinants of health, there is great potential to develop community-informed interventions that promote well-being in KwaZulu-Natal as well as in other areas with similar populations.

### Current status

Despite the COVID-19 pandemic, in wave 3 the Asenze team has assessed 80% of the wave 2 participants. The arrival of the pandemic in the midst of wave 3 allows us to investigate the impact of COVID-19 on the cohort, as a proportion of the children were assessed before and the rest after the COVID-19 pandemic began. The wave 3 data collection will also capture ways in which the pandemic has affected poorly resourced communities and vulnerable populations in South Africa and can be of value in making policy recommendations.

## DATA ACCESSIBILITY

The study data are not freely available online, but MPIs Drs. Desmond and Davidson would welcome collaborations with other researchers and data sharing is possible upon request and ethics approval. For further information, contact Chris Desmond at ChrisDesmond@libstudies.com.

## Figures and Tables

**Table 1.** Measures by wave and by respondent

Category	Measure	Description	Respondent	W1	W2	W3
Demographics	Caregiver demographics^1^	Date of birth, gender/sex, education, work status, marital status, etc.	Caregiver	X	X	X
	Parent whereabouts^1^	Location/status of father and mother	Caregiver	X		X
	Child demographics^1^	Date of birth, gender/sex, education, support grants, etc.	Caregiver	X	X	
	Household characteristics^2^	Household composition/structure, sanitation access, unemployment/income status, etc.; Adapted from the South African Demographic and Health Survey, 2003	Caregiver	X		X
Child cognitive development	Grover Counter Scales^2^	Assesses cognitive functioning independent of verbal/ language abilities	Child	X	X	
	Kaufman Assessment Battery for Children (ABC) subscales: Hand Movements^3^, Atlantis^3^, Atlantis Delayed^3^	Assesses cognitive functions of attention, concentration, and memory	Child	X	X	X
	Kaufman ABC Conceptual Thinking^3^	Assesses abstract reasoning and visual perception through simultaneous processing	Child	X		
	Kaufman ABC Rover for >6 yr age group^3^	Assesses executive functioning	Child		X	X
	Kaufman ABC planning^3^	Assesses executive functioning problem solving through pattern reasoning	Child			X
	Neuroscreen Processing Speed Test, Trail Making Test, and Digit Span Test^3^	Assesses executive function processing speed, visual attention and sequencing, and working memory	Child			X
	Reynell Developmental Language Scales^3^	Assesses expressive and receptive language	Child	X		
	Hopkins Verbal Learning^3^	Assesses verbal learning and memory	Child		X	
Child health, behavioral functioning, and achievement	Ten Question Screener^2,3^	Screens for serious cognitive, motor, seizure, behavior, and other neurodevelopmental disabilities	Caregiver	X		
	McCarthy Scales of Children’s Ability^3^	Assess cognitive motor abilities	Child	X	X	
	World Health Organization Disability Assessment Schedule^3^	Assesses problems with functioning due to health conditions (e.g., disease, injuries, mental/ emotional problems, drug/alcohol problems)	Child			X
	Child Medical Assessment Form^1^	Assesses medical history	Caregiver	X	X	
	Height, weight, vision, hearing^3^	Height obtained from a scale; Weight obtained from a stadiometer; Vision assessed with the Snellen E chart; Hearing assessed using otoacoustic emissions/ audiometry	Caregiver and Child	X	X	X
	Hemoglobin, HIV, immunity history, anemia^3^	Voluntary counseling and rapid testing used for HIV; HemoCue used to test hemoglobin	Caregiver and Child	X	X	X
	The Strengths and Difficulties Questionnaire^3^	Assesses emotional and behavioral functioning with subscales: emotional, conduct, hyperactivity, peer relationship problems, prosocial behavior; More information: www.sdqinfo.org	Caregiver	X	X	X
			Teacher		X	
			Adolescent			X
	Adapted Behavior Assessment System Subscales^3^	Assesses adaptive functioning	Caregiver	X		
	Takalani-Sesame: Literacy Subtest	Assesses child literacy	Child		X	
	Wide Range Achievement Test ^3^: arithmetic sub-test	Assesses numeracy	Child		X	
	Obedience and respect^1^	Assesses caregiver’s perception of child’s obedience and respect	Caregiver			X
Adolescent psychosocial and other	Pubertal Development Scale^3^	Assesses physical growth and development via self-report	Adolescent			X
	Pregnancy^1^	Assesses current or past pregnancies/ pregnancy outcomes; Adapted from a HIPSS	Adolescent			X
	Respondents living with HIV^1^	Assesses HIV status and treatment; Adopted from HIPSS	Adolescent			X
	The Short Form Health Survey^2^	Assesses physical and mental health functioning via self-report	Adolescent			X
	National Survey on Drug Use and Health Questionnaire^3^	Assesses initiation, frequency, intoxication with, and cravings for alcohol, cigarettes, and other substances	Adolescent			X
	Patient Health Questionnaire 9-item Scale^3^	Assesses depression	Adolescent			X
	Generalized Anxiety Disorder 7-item Scale^3^	Assesses anxiety	Adolescent			X
	Sexual history	Assesses sexual behavior (e.g., time of initiation, partner age, condom use); Adapted from HIPSS and the DREAMS	Adolescent			X
	Exposure to community violence^3^	Assesses exposure to community violence; adapted from the US CDC Youth Risk Behavior Survey	Adolescent			X
	Resistance to Peer Influence Scale^3^	Assesses ability to resist peer pressure	Adolescent			X
	Retrospective Bullying Questionnaire^3^	Assesses experiences of bullying as victim; Adapted from CDC	Adolescent			X
	Deviant Peers Measure	Assesses deviant behaviors of peers; Adapted from the Youth Health and Prevention Project	Adolescent			X
	Exposure to violence^3^	Assesses exposure to physical, sexual, and/or intimate partner violence; Adapted from the US CDC 2019 Youth Risk Behavior Survey	Adolescent			X
	Desmond risk experiment game^1^	Assesses attitude to risk taking; adapted from the Binswanger Attitudes towards Risk Experiment	Adolescent			X
	Barratt Impulsivity Measure: 8 Item version^3^	Assesses impulsivity	Adolescent			X
	Adapted Gender Equitable Measurement Scale^1^	Assesses perception of gender roles and equity	Adolescent			X
	SUUBI Social Support Behaviors^3^	Assesses perception of family support	Adolescent			X
	Short Grit Scale^3^	Assesses perseverance	Adolescent			X
	Community support	Assesses perception of community support; Adapted questions from the Youth Health and Prevention Project	Adolescent			X
	Hope and future orientation^1^	Assesses beliefs and expectations adolescent’s hold about their future	Adolescent			X
	Media use^1^	Assesses use of phone/Internet/TV	Adolescent			X
	Employment	Assesses past/current employment; Adapted from Cape Area Panel Study	Adolescent			X
	Education^1^	Assesses education, including school enrollment and current grade	Adolescent			X
	Gangs and perpetration of violence^1^	Assesses exposure to or participation in gang activity	Adolescent			X
	Psychological Sense of School Membership Scale^2^	Assesses school experience	Adolescent			X
	Exposure to previous interventions^1^	Assesses exposure to school or community education programs (e.g., sexual health, substance use)	Adolescent			X
	Post-survey feedback	Assesses experience completing W3; Adapted from the Child and Adolescent Self-Awareness and Health 3 study	Adolescent			X
	COVID-19^1^	Assesses experience during COVID-19 (e.g., concerns of infection, school and socializing during lockdown)	Adolescent			X
Family functioning and environment	Parenting Stress Index^3^	Assesses caregiver stress, burden, role strain, and competence	Caregiver	X	X	X
	Trends in Maths and Science	Assesses caregiver educational aspirations for children	Caregiver			X
	Confusion, Hubbub, and Order Scale^3^	Assess household confusion and disorganization	Caregiver	X	X	X
	Family Stress and Trauma Questionnaire	Assesses economic/material deprivation and exposure to violence/abuse	Caregiver	X	X	X
	Food security^1^	Assesses household food security; Adapted questions from DREAMS	Caregiver			X
	Alcohol use^1^	Assesses household alcohol use	Caregiver		X	X
	Home Observation for Measurement of the Environment (HOME)^3^	Assesses household environment; Adapted from HOME Inventory	Caregiver	X	X	
Caregiver functioning and psychosocial	Client Diagnostic Questionnaire^2,3^	Screens for psychiatric disorders: depression, PTSD, substance abuse; Adapted from Primary Care Evaluation of Mental Disorders Screen	Caregiver	X	X	X
	Alcohol Use Disorders Identification Test^2^	Screens for alcohol misuse, initiation, and frequency	Caregiver	X	X	X
	Partner violence^2^	Screens for experience with partner violence; Adapted from the South African Medical Research Council	Caregiver	X	X	X
	The Short Form Health Survey^2^	Assesses physical and mental health functioning via self-report	Caregiver	X	X	X
	NeuroScreen Processing Speed Test, Trail Making Test, and Digit Span Test^3^	Assesses executive function processing speed, visual attention and sequencing, and working memory	Caregiver			X
	Hemoglobin, HIV, immunity history, anemia^3^	Voluntary counseling and rapid testing used for HIV; HemoCue used to test hemoglobin	Caregiver	X	X	X
	HIV and pregnancy	Assesses HIV and pregnancy status; Adapted questions from the HIPSS	Caregiver			X
	Short HIV stigma scale^3^	Tests HIV positive caregiver for experience/perceptions of HIV stigma	Caregiver			X
	Social Support Behaviors Scale Family Social Support Questionnaire^3^	Assesses emotional support, socializing, practical/ financial assistance, and advice/ guidance	Caregiver	X	X	X
	Exposure to interventions	Assesses exposure to community education interventions (e.g., parenting, subsidies); Adapted from DREAMS	Caregiver			X
	COVID-19^1^	Assesses experience during COVID-19 (e.g., concerns, employment, access to services/testing, food security)	Caregiver			X

W, wave; COVID-19, coronavirus disease 2019; HIPSS, Center for the AIDS Programme of Research in South Africa HIV Incidence Provincial Surveillance System; DREAMS, United States Agency for International Development Partnership Determined, Resilient, Empowered, AIDS-free, Mentored and Safe; CDC, Centers for Disease Control and Prevention, United States; PTSD, post-traumatic stress disorder; HIV, human immunodeficiency virus; AIDS, acquired immune deficiency syndrome.

1 Developed or adapted for Asenze.

2 Validated measure in South Africa.

3 Validated measure.

**Table 2.** Household demographics and family context during wave 1 (n=1,382 households; 1,581 children)

Variables	n (%)	Missing, n (%)
No. of children per household enrolled in Asenze, [Min-Max]	[1-3]	0 (0.0)
No. of children (age <18) in household, mean±SD [Min-Max]	3.6±1.9 [1-16]	38 (2.8)
No, of adults (age 18+) in household, mean±SD [Min-Max]	3.7±2.0 [1-13]	39 (2.8)
Amount spent on food in past month (South African Rand), mean±SD [Min-Max]	803±474 [100-4,000]	115 (8.3)
Employed adult(s) in household	1,045 (75.6)	49 (3.6)
Household member with education past high school	120 (8.7)	47 (3.4)
Recent (past 2 yr) death of a household member	297 (21.5)	9 (0.7)
One or more children in household on social grants	945 (68.4)	45 (3.3)
Household ran out of food during past month	321 (23.2)	71 (5.1)

SD, standard deviation; Min, minimum; Max, maximum.

**Table 3.** Children’s characteristics during wave 1 (n=1,581 children)

Characteristics	n (%)	Missing, n (%)
Age, mean±SD [Min-Max] (yr)	5.0±0.6 [3.7-6.6]	0 (0.0)
Birthweight, mean±SD [Min-Max] (kg)	2.9±0.6 [0.9-5.3]	197 (12.5)
Female	791 (50.0)	0 (0.0)
Received preschool education (daycare or crèche)	967 (61.2)	6 (0.4)
Ever breastfed	1,286 (81.3)	47 (3.0)
Maternal alcohol use during pregnancy	105 (6.6)	102 (6.5)
Mother’s whereabouts		24 (1.5)
Living with child	1,214 (76.8)	
Not living with child, employed elsewhere	194 (12.3)	
Not living with child, ill in hospital	4 (0.3)	
Deceased	94 (6.0)	
Absconded	51 (3.2)	
Father’s whereabouts		28 (1.8)
Living with child	496 (31.4)	
Not living with child, employed elsewhere	546 (34.5)	
Not living with child, ill in hospital	19 (1.2)	
Deceased	135 (8.5)	
Absconded	355 (22.5)	
In jail	2 (0.1)	
Mother’s education level		41 (2.6)
None	65 (4.1)	
Grade 1-7 (primary)	223 (14.1)	
Grade 8-11 (high school)	665 (42.1)	
Grade 12 (matric)	359 (22.7)	
>Grade 12 (college/tertiary)	78 (4.9)	
Unknown	147 (9.3)	
Father’s education level		31 (2.0)
None	88 (5.6)	
Grade 1-7 (primary)	170 (10.8)	
Grade 8-11 (high school)	407 (25.7)	
Grade 12 (matric)	437 (27.6)	
>Grade 12 (college/tertiary)	64 (4.1)	
Unknown	382 (24.2)	
Child’s HIV status^1^		0 (0.0)
Positive	62 (3.9)	
Negative	1,278 (80.8)	
Indeterminate test results	4 (0.3)	
Unknown status and declined testing	237 (15.0)	

SD, standard deviation; Min, minimum; Max, maximum; HIV, human immunodeficiency virus.

1 Composite variable based on rapid HIV test results and/or primary caregiver report of child’s status.

**Table 4.** Primary caregivers’ characteristics during wave 1 and wave 2

Characteristics	Wave 1^1^	Missing	Wave 2^2^	Missing
Age, mean±SD [Min-Max] (yr)	36.9 ±13.8 [16-88]	357 (24.8)	38.9±13.5 [15-88]	0 (0.0)
Sex				
Female	1,379 (96.0)	28 (2.0)	1,242 (98.0)	0 (0.0)
Primary caregiver type				
Mother	1,037 (65.6)	0 (0.0)	870 (61.8)	0 (0.0)
Father	24 (1.5)		15 (1.1)	
Grandmother	303 (19.2)		336 (23.9)	
Other	217 (13.7)		188 (13.3)	
Highest education level				
None	63 (4.4)	753 (52.4)	Not collected	Not collected
Grade 1-7 (primary)	154 (10.7)		Not collected	
Grade 8-11 (high school)	291 (20.3)		Not collected	
Grade 12 (matric)	132 (9.2)		Not collected	
>Grade 12 (college/tertiary)	26 (1.8)		Not collected	
Unknown	18 (1.3)		Not collected	
Ever experienced intimate partner violence	619 (43.1)	7 (0.5)	588 (46.2)	115 (9.0)
Cigarette smoker (past 6 mo)	46 (3.2)	3 (0.2)	31 (2.4)	8 (0.6)
Hazardous alcohol consumption (AUDIT score ≥8)	44 (3.1)	67 (4.7)	52 (4.1)	7 (0.6)
Binge drinking (AUDIT-consumption score ≥3 for female or ≥4 for male)	126 (8.8)	65 (4.5)	102 (8.0)	7 (0.6)
Mental health disorder, any (Client Diagnostic Questionnaire)	449 (31.3)	11 (0.8)	240 (18.9)	119 (9.4)
HIV status^3^				
Positive	377 (26.2)	0 (0.0)	401 (31.5)	0 (0.0)
Negative	932 (64.9)		815 (64.0)	
Unknown	128 (8.9)		57 (4.5)	

Values are presented as number (%). SD, standard deviation; Min, minimum; Max, maximum; AUDIT, Alcohol Use Disorders Identification Test; HIV, human immunodeficiency virus.

1 Caregivers: 1,437 and children: 1,581.

2 Caregivers: 1,273 and children: 1,409.

3 Composite variable based on rapid HIV test results and/or primary caregiver self-report of HIV status.
